# Nature of Job and Psychiatric Problems: The Experiences of Industrial Workers

**DOI:** 10.5539/gjhs.v7n1p288

**Published:** 2014-10-09

**Authors:** Syed Khalid Perwez, Abdul Khalique, H. Ramaseshan, T. N. V. R Swamy, Mohammed Mansoor

**Affiliations:** 1 VIT Business School, VIT University, Vellore-632014, Tamil Nadu, India; 2 P.G Department of Psychology, Ranchi University, Ranchi, India

**Keywords:** psychiatric problems, nature of jobs, industrial workers

## Abstract

**Aim:**

The present study aimed to examine the effect of nature of job (High risk/low risk) on psychiatric problems of 200 workers of Tata Motors Ltd. in Jamshedpur. The workers/participants were divided on the basis of the nature of their job (high/low risk) and their salary (high/low paid) resulting in four sub-groups with 50 participants respectively s.

**Methods:**

The Middlesex Hospital Questionnaire (M.H.Q) constructed by Crown and Crisp (1966) and adapted in Hindi by Srivastava and Bhat in 1974 was administered on the participants.

**Results:**

Results clearly indicated that nature of job (high and low risk) played a significant role in creating psychiatric problems in workers. Workers doing high risk jobs showed a greater amount of psychiatric problems compared to workers doing low risk jobs in both high paid and low paid categories. Psychiatric problems included free-floating anxiety, obsessional traits and symptoms, phobic anxiety, somatic concomitants of anxiety, neurotic depression, and hysterical personality traits were seen more in high risk job workers.

**Conclusions:**

High risk job workers had significantly higher psychiatric problems compared to low risk job workers.

## 1. Introduction

Research on the health impact of job conditions has long been a concern of occupational medicine, and attention has focused on physical and chemical hazards. Recently, psychological and social factors related to technology and job content have been identified as possible risk factors for the health and well being of workers. This has extended the “work-environment” concept, which attempts to identify the psychological problems and nature of job relationship with the health and psycho-social outcomes of the workers.

India has witnessed rapid industrialization in the past four decades and millions have been employed in various industries. It has been established that industrialization and urbanization give rise to various mental health problems in industrial employees and that thses problems adversely affect the mental health of individual employees and thereby may negatively interfere withe productivity and economy.

Mental health deals with psychology of normal working people, not only with psychiatric causes, but it also focuses on an overall assessment of the mental health. The concept of mental health is taken in a wider sense. It is not a representation of any psychodynamic unit but as a loose description, designated for an overall level of success, personal satisfaction, effectiveness and excellence of the individual’s functioning.

Mental health can be defined as the ability to balance feelings, desires, ambitions, and ideals in daily life. It means the ability to face and accepts the realities of life (Kuppuswami, 1962). According to Kornhauser (1965) mental health also refers to a combination of psychological and behavioral attributes, some of which the person must possess above a required minimum, and others of which signify better mental health the more they are present. Higher index of mental health indicates high individual effectiveness, and good effectiveness reflected in organizational effectiveness. With advancement of industrialization and urbanization in our country, the problem of mental health among workers/employees is growing in importance. The burden of mental disorders on health and productivity throughout the world has long been profoundly underestimated (US Surgeon General, 2000). The impact of mental health problems in the work place has serious consequences not only for the individuals whose lives are influenced either directly or indirectly, but also for enterprise productivity. Mental health difficulties strongly influence the employee’s performances, rates of illness, absenteeism, accidents and staff turnovers.

Some employees at their jobs in the factory look feel and behave differently as compared to others. They will often appear bored, irritated, frustrated and tense, probably grumbling about the management and perhaps watching the clock until they can leave. Some may be political extremists, others just the rather unhappy people, who are to be found in every industry. In the factory strikes, absenteeism, illness and psychosomatic breakdown is unduly common. These are the keys to industrial mental health and to a high level of productivity in all industrialized countries. Moreover, these minor psychological symptoms usually remain unnoticed and so do not receive professional assistance. The practical importance of these symptoms lies in the fact that not only they lead to impairment in productivity, but also mistakes at work and accident proneness.

### 1.1 Literature Review

The pioneer scientific study in relation to mental health and distress of individual workers was conducted by Kornhauser in 1964. He found that a worker’s performance at his task is closely related to his mental health. He studied the psychological condition of workers in modern mass production industries and attempted to assess and compare the mental health of men at higher and lower skill levels, with special attention to the human effects of a routine production job.

Psychiatric symptoms could be the result of the increased stress levels at work as reported by Sauter et al. (1990) and Khalique and Khalid (2009). Dysfunctional interpersonal relationship increased job pressure, greater responsibility without authority and feeling of insecurity, nature of job like high and low risk job (Khalid & Khalique, 2012) have been reported as the sources of stress. Career problem and pressure for production are also known to increase stress.

Srivastava (1983) examined the effect of perceived role stress, resulting from role ambiguity, role conflict and role overload on mental health on a sample of white-collar employees (N=200). The investigation revealed that the perceived role stress was found to be positively associated with various criteria of mental ill-health, such as free floating anxiety, obsessive compulsive neurosis, phobic anxiety, somatic concomitant anxiety, neurotic depression and hysterical traits and symptoms.

Nanda, Das and Mishra (1989) studied two different types of working environment at Rourkela Steel Plant-one was [past tense was] the Iron and Steel zone, considered as hazardous, and the other, the Mill and Services zone, considered as less hazardous. After comparing the psychiatric morbidity from these two areas it was found that the psychiatric morbidity was higher in the hazards areas, especially for neurosis and alcoholism.

Kar and Haridas (2003) observed that anxiety symptoms such as inner tension, worrying, concentration difficulty, indecision, and depressive symptoms such as sad mood, fatigability, pessimistic thought and inability to feel were reported by more than 5% of industrial employees. Somatic symptoms such as reduced sleep, decreased appetite, reduced sexual drive, aches and pains, muscular tension were also very common. A considerable proportion had hostile attitudes.

Dewa et al. (2007) studied the links between psychiatric disorders and work-related stress as well as between psychiatric disorders and physical conditions. The study explored the relationships between chronic work stress, psychiatric disorders, and chronic physical conditions and disability among workers. By doing so, the study sought to understand how these factors were associated with worker disability when they were experienced alone versus in combination with one another.

It is evident from the above discussion that nature of job stress is one central key ‘cause’ of psychiatric problems in industrial workers. But there is great dearth of such studies in Indian situation.

Against this background, the present study was done with the following three objectives in view:

Ø To study the effect of nature of job (high risk and low risk job workers) on psychiatric problems.
Ø To examine the probability of psychiatric problems of industrial workers.
Ø To find out the important symptoms that cause psychiatric problems among the high and low risk industrial workers.

### 1.2 Hypothesis

To meet the above objectives, the following hypothesis had been formulated:

$$$

Ø The high risk job workers will show more psychiatric problems compared to low risk job workers.

## 2. Methodology

### 2.1 Sample

A sample of 200 workers was selected from the TATA Motors Ltd., Jamshedpur. The workers were divided into two categories namely ‘High Risk’ workers and ‘Low Risk’ participants and further, these workers were sub-divided into two categories, that is, ‘Low Paid’ and ‘High Paid’ participants. The sample was based on a 2×2 factorial design. Therefore, there were four sample sub-groups and each sub-group was represented by 50 respondents, making a total of 200. The sample design is given below:

### 2.2 Sample Design

**Table:** 

Remuneration	Nature of Job	

	High Risk Job Workers	Low Risk Job Workers
High Paid	50	50
Low Paid	50	50
Total	200	

### 2.3 Tools/Instruments Used

The following tools/research instruments have been used in the present study for the collection of data:

#### 2.3.1 Personal Data Sheet

Personal Data Sheet was specially designed for the present study which include data related to personal identification of the workers, specially their names, addresses, factories in which they are working, designation, nature of the job and salary etc.

#### 2.3.2 The Middlesex Hospital Questionnaires (M.H.Q.)

The presence of mental ill health of the Tata Motors Workers was measured by using the Hindi version of the Middlesex Hospital Questionnaire. The Middlesex Hospital Questionnaire is a self rating scale. It is a short and simple scale which takes hardly 7-10 minutes for administration. It is constructed by Crown and Crisp (1966) and adapted in Hindi by Srivastava and Bhat in 1974. This test provides a rapid quantification of common symptoms and traits relevant to the conventional diagnostic categories of neurotic illness. This test consists of six sub-scales having 8 questions each. These sub scales are: Free-floating anxiety (FFA), Obsessional traits and symptoms (OBS), Phobic anxiety (PHO), Somatic concomitants of anxiety (SOM), Neurotic depression (DEP) and Hysterical personality traits (HYS).

#### 2.3.3 Statistical Analyses

The following statistical techniques have been used in the analysis of obtained data:

- As the data is based on a 2×2 factorial design, the analysis of variance (ANOVA) has been relied upon to examine the effect of factors.
- The sub-groups based on the nature of jobs have been compared by t-test.
- The mean scores have also been calculated and graphically illustrated.

## 3. Findings and Discussions

An attempt has been made to examine the relationship of nature of jobs (high-low risk) with psychiatric problems and its sub-scales of MHQ. The psychiatric illnesses have been measured by the Hindi version of Middlesex Hospital Questionnaire (MHQ) adapted by Srivastava and Bhatt (1974). As mentioned earlier in the discussion the participants s have been classified in two categories depending on the levels of risk and levels of payment in their jobs. To examine the status of psychiatric problems of both categories of workers mean scores, ANOVA, t-test have been taken into account.

The statistical significance of mean scores of high risk and low risk job workers was examined. As the sample is based on 2×2 factorial design, ANOVA has been calculated to examine the role of nature of job and their interaction effect of psychiatric problems on MHQ and its sub-scales. Table 1 presents the F-ratios on MHQ and its sub-scales.

**Table 1:** Analysis of variance of scores on Middlesex Hospital Questionnaire (MHQ) and its sub-scales: Risk Factors

Source of Variation Risk Factors 6 - SUB SCALES OF M.H.Q.		SUM OF SQUARES	df	F - ratio
I.	Free Floating Anxiety (FFA)	266.805	1	45.127**
II.	Obsession Compulsion (OBS)	231.125	1	43.116**
III.	Phobia (PHO)	151.380	1	31.768**
IV.	Somatic Complaints (SOM)	292.820	1	38.786**
V.	Depression (DEP)	327.680	1	51.410**
VI.	Hysteria (HYS)	184.320	1	39.137**
	TOTAL M.H.Q.	8580.500	1	47.542**

** = Significant at above .01 level.

The Table 1 reveals that the two groups show statistically significant higher psychiatric symptoms. For this purpose the means scores of both the groups on M.H.Q. and its sub- scales have been presented in Table 2.

**Table 2:** Mean scores of High Risk & Low Risk Job workers on M.H.Q. and its sub-scales

6 SUB SCALES OF M.H.Q.	
		HIGH RISK	LOW RISK
		N = 100	N = 100
		MEAN	MEAN
I.	Free Floating Anxiety (FFA)	8.4	6.09
II.	Obsession Compulsion (OBS)	6.47	4.32
III.	Phobia (PHO)	5.35	3.61
IV.	Somatic Complaints (SOM)	7.59	5.17
V.	Depression (DEP)	7.08	4.52
VI.	Hysteria (HYS)	5.93	4.01
	TOTAL M.H.Q.	40.82	27.75

Table 1 reveals that all the F-values between the workers of high and low risk jobs pertaining to six sub-scales of M.H.Q are statistically significant at above .01 levels. Considering the significant F-values, the mean scores of both the high risk and low risk job workers have been calculated and presented in Table 2.

It can be seen that the mean scores of high risk job workers are comparatively much higher than low risk job workers in all the sub scales of M.H.Q as well as in total M.H.Q. There is a clear indication that workers engaged in high risk jobs have more psychiatric difficulties as compared to employees in low risk job.

**Figure 1:**
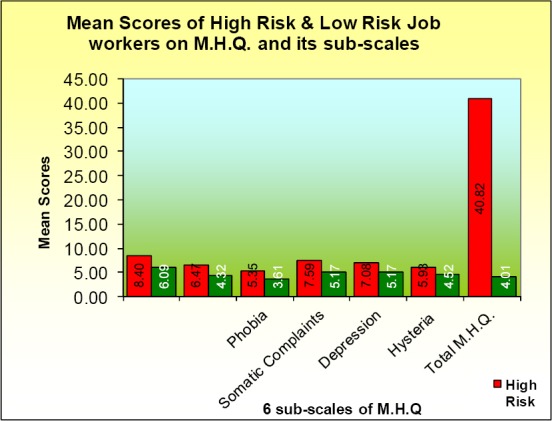
Mean scores of high risl & low risk job workers on M.H.Q.and its sub-scales

For the visual presentation of the mean scores reported in the above table Figure 1 has been plotted which clearly shows the trend reported above. The bars of high risk job worker are higher than those of low risk job workers.

Thus, it can be concluded that the high risk job workers have high free floating anxiety, obsession compulsion, phobia, somatic complaints, depression and hysteria as compared to low risk job workers.

Considering the significance levels of F-ratios on M.H.Q. and its sub-scales the sub groups of high risk and low risk job workers have also been compared through t-tests. These comparisons have been done separately for high paid and low paid job workers. Table 3 presents the comparisons of high risk and low risk job workers in high paid category. The table contains the mean scores, S.Ds and t-values comparing workers in two different groups (high/low risk).

**Table 3:** Comparisons of High Risk and Low Risk Job workers in High Paid Category on M.H.Q. and its sub-scales: t values

6 SUB SCALES OF M.H.Q.	
		HIGH RISK		LOW RISK	
		N = 50		N = 50	
		MEAN	S.D.	MEAN	S.D.	t-value
I.	Free Floating Anxiety (FFA)	7.76	2.61	5.88	2.47	3.69**
II.	Obsession Compulsion (OBS)	5.96	2.41	4.32	2.35	3.42**
III.	Phobia (PHO)	5.22	2.04	3.44	2.17	4.34**
IV.	Somatic Complaints (SOM)	7.36	2.94	4.9	2.67	4.39**
V.	Depression (DEP)	6.6	2.52	4.32	2.34	4.65**
VI.	Hysteria (HYS)	5.54	2.41	3.9	1.94	3.64**
	TOTAL M.H.Q.	38.44	14.O7	26.82	13.06	4.27**

** = Significant at above .01 level.

There are 7 t-values and all the t-value are statistically significant (above .01 level of significance). This indicates that the nature of jobs (high risk and low risk) plays a significant role in the development of psychiatric symptoms. Further the mean scores of high risk and low risk jobs workers have shown that in all the 7 comparisons of M.H.Q and its sub-scales the mean scores of high risk jobs workers are significantly higher than those of low risk job workers. This trend has been marked in the case of high paid category.

Similar comparisons of high risk and low risk job workers have also been made for low paid job workers. The mean scores, SDs and t-values are given in Table 4.

**Table 4:** Comparisons of High Risk and Low Risk Job workers in Low Paid Category on M.H.Q. and its sub-scales: t values

6 SUB SCALES OF M.H.Q.	
		HIGH RISK		LOW RISK		
		N = 50		N = 50		
		MEAN	S.D.	MEAN	S.D.	t-values
I.	Free Floating Anxiety (FFA)	9.04	2.23	6.3	2.39	5.83**
II.	Obsession Compulsion (OBS)	6.98	2.42	4.32	2.06	5.91**
III.	Phobia (PHO)	5.48	2.43	3.78	2.07	3.69**
IV.	Somatic Complaints (SOM)	7.82	2.77	5.44	2.60	4.41**
V.	Depression (DEP)	7.56	2.74	4.72	2.48	5.46**
VI.	Hysteria (HYS)	6.32	2.43	4.12	1.84	5.00**
	TOTAL M.H.Q.	43.2	14.02	28.68	12.66	5.44*

** = Significant at above .01 level.

In the table referred above it has been observed that all the 7 t-values of M.H.Q and its sub-scales are statistically significant at above 0.01 levels of significance. This indicates that nature of job (high risk and low risk) plays a significant role in creating psychiatric problems in low paid workers also. Further the mean scores of high risk and low risk job workers have shown that in all 7 comparisons of M.H.Q. and its sub-scales high risk job workers have obtained significantly higher mean scores compared to those of low risk job workers in low paid job category also. This clearly indicates that high risk job workers have more psychiatric problems than low risk job workers. Data of both the high and low paid workers presented in above referred tables and figures confirm the finding that the high risk job workers have higher psychiatric problems than the low risk job workers.

## 4. Conclusions

Above analysis, finding and discussion have been made to examine the relationship of nature of jobs (high risk and low risk) with psychiatric problems. It may be recalled that a hypothesis has been formulated that ***“high risk job workers will show more psychiatric problems compared to low risk job workers”*.**

Let the hypothesis be examined in the light of analysis and discussions reported above. The F-ratio (Table 1) has indicated the significant role of the nature of jobs on psychiatric problems. The high risk job workers have shown significantly higher or more psychiatric problems as compared to low risk job workers (Table 2 and Figure 1). The high risk and low risk job workers have also been compared with t-tests separately for high paid groups (Table 3) and low paid group (Table 4). All the t-values reported in two tables have indicated that nature of job (high risk and low risk) plays a significant role in creating more psychiatric problems in workers doing high risk jobs compared to low risk jobs.

In the light of the above findings it can clearly be concluded that hypothesis formulated in relation to nature of jobs and psychiatric problems has been fully supported and confirmed.

The present research is a modest attempt to study nature of psychiatric problems among the industrial workers. These findings have important policy implications. They suggest that psychiatric problems in the work place have adverse effect on productivity. The industries should make policies for creating congenial atmosphere and to manage risk through interventions like job rotations, providing adequate rest intervals etc. with lesser psychiatric difficulties to enhance their productivity.
